# The impact of cognitive biases, mental models, and mindsets on leadership and change in the health system

**DOI:** 10.1177/08404704231215750

**Published:** 2023-11-27

**Authors:** David A. Petrie, Ronald R. Lindstrom, Samuel G. Campbell

**Affiliations:** 1 12361 Dalhousie University, Halifax, Nova Scotia, Canada.; 2 8166 University of British Columbia, Vancouver, British Columbia, Canada.

## Abstract

Understanding how cognitive biases, mental models, and mindsets impact leadership in health systems is essential. This article supports the notion of cognitive biases as flawed thinking or cognitive traps which negatively influence leadership. Mental models that do not fit with current evidence limit our ability to comprehend and respond to system issues. Resulting mindsets affect cognition, behaviour, and decision-making. Metacognition is critical. The wicked problems in today’s complex health system require leaders and everyone involved to elevate their personal, organizational, and disciplinary perspectives to a systems level. Three examples of mental models/mindsets are reviewed. They do not change simply because we wish or will them to. The first step is being aware of what they are and how they impact our thinking and decision-making. Some tips for managing these traps are offered as examples of how to challenge our leadership approach in the health system.

## Introduction

### We are trying to operate on old ideas and systems that are clearly inadequate to the present crisis - Kim Stanley Robinson

Health systems in Canada are in crisis.^1^ They were in trouble before the COVID-19 pandemic and did not pass the stress test as described in the aptly titled article “The Health System is on Fire—and it was Predictable.”^2^ Much has been written about direct causes (too much), and root causes (not enough), and their potential solutions,^3,4^ but there is still an under-emphasis on the role of biases, mental models, and mindsets in health leadership and system transformation.^5,6^

**Figure 1.**
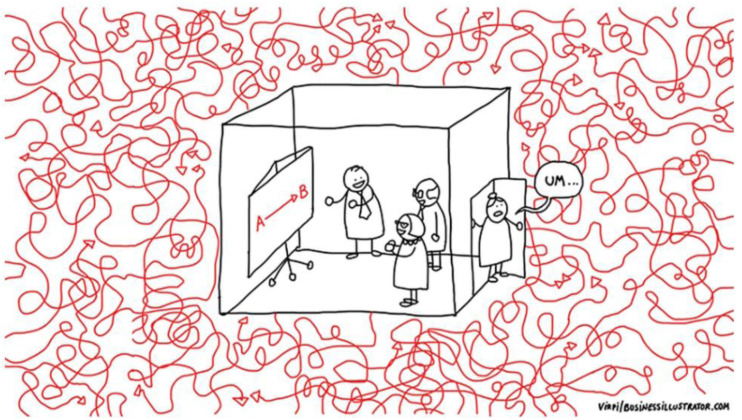
Complexity outside linear office.

Conventional thinking and fixed mindsets are no longer fit for purpose (if they ever were). Getting stuck in these old assumptions and cognitive biases^7-9^ has led to “unstable, uncontrollable situations”^7^ and seemingly intractable problems in healthcare (Figure 1). Einstein famously said: “how we formulate the problem is far more essential than the solutions.” How we formulate problems and make sense of our world (and therefore, make decisions) depends upon our biases, mental models, and mindsets.^10^

Catalyzing and influencing, let alone leading, change is difficult in complex systems. Rarely, if ever, do we see “transformational change” in such systems driven and managed from above. So how does large-scale change^9^ happen, and how can we lead it in healthcare systems? The simple answer is that change happens in many different and unpredictable ways… but that does not mean that we cannot play an important role in the emergence of a better system. Understanding how biases, mental models, and mindsets can impact leadership and foster change in health systems is essential.^11^

## Say what?

### The illiterate of the 21^st^ century will not be those that cannot read and write, it will be those with the inability to learn, unlearn, and re-learn - Alvin Toffler

There is some debate about the academic definitions of cognitive biases, mental models, and mindsets and conflation and confusion around the relationships between the concepts. Some would argue that they are similar, or even synonymous. Others would argue that there are important distinctions. The intent of this brief overview is to reintroduce the importance of these concepts in the context of our system’s need to evolve (perchance transform) to meet the needs of the populations we serve. This is not a comprehensive literature review or meta-analysis meant to finalize our positions; rather, it is an exploratory reflection to stimulate more interest from readers.

First, for our purposes, we see cognitive biases as flawed thinking or cognitive traps of which we are unaware^11^ and which can negatively affect decision-making. Table 1 lists some examples of common biases/traps and examples of how they may impact health systems. As found in a recent systematic literature review^11^ examining biases and decision-making particularly during times of major transformation, managing cognitive biases is critical to the success of strategic decisions.

**Table 1.** Some common cognitive biases and examples in the health system^5,10-12^.

Bias	Working definition	Examples from health system leadership
Anchoring bias	Relying too much on initial thoughts	Focusing on a crowded emergency department as a place to direct attention to, when a closer look would have identified downstream access block and flow issues as the appropriate target
Confirmation bias	Finding evidence that supports your beliefs and ignoring what doesn’t	Judging decreased complaints as an indicator of improved quality without noticing that the new complaint process is unwieldy
Over-confidence bias	Overestimating of your abilities; (aka Dunning-Kruger effect)	Allowing the leader of a prior initiative to overly influence the direction and decisions for a problem in a different context
False consensus bias	Overestimating how much others agree with you	An overbearing or intimidating leader presents his case and takes silence from his team as agreement
Sunk-cost bias	Protecting earlier investments/choices	A politically prominent and publicly championed “system innovation” is found to have made no difference to patient experience or population outcomes in a well-done evaluation, yet funding is renewed for another year
Blind spot bias	Recognizing biases in others but not yourself	Accusing another of self-interest in supporting a “pet” project, but blindly doing the same thing in another context

Second, mental models are an internal representation of external reality.^13^ They are often constructed, reflected upon, and iterated/improved (and distorted) over time though we can adopt and adapt mental models without questioning them through the culture and discourse of our group. In the context of systems thinking,^14,15^ humans “don’t interact with reality directly…and indirectly relate to reality through our mental models of it.”^14^ This notion is reinforced as three essential “truths” outlined by Meadows:^14^ “Everything we think we know about the world is a model; our models usually have a strong congruence with the world; however, and conversely, our models fall far short of representing the world fully.” Or, as George Box said: “All models are wrong, but some are more useful than others.”

Third, we concur with the characterization of mindsets as a collection or set of mental models.^16,17^ Mindsets directly affect cognition, behaviour, and decision-making. More importantly, the practical consequences of not intentionally reflecting upon one’s mindset can impact sense-making in unexpected or inimical ways.

For the purposes of this article, we will leave the debate about the nuances of these theories and concepts to the academics and ask the reader to hold the terms lightly, but we encourage the reader to inquire into these concepts. The important point is summed up by Max Plank: “If you change the way you look at things, the things you look at change.”

Making sense of our reality is essential to making good decisions. And the health system is a product of the many decisions made by various agents (patients, clinicians, leaders, organizations, governments) over time and across scales. Shared and functional mental models can help individuals, teams, and organizations work towards a common purpose (like the Quintuple Aim^18^). Flawed mental models, or those that no longer fit the evidence and scrutiny of pragmatic/real-world experience, limit our ability to respond to system issues. Put simply, if the mental model we have of our world is that it is flat, our explorers and innovators will avoid horizons, and we will have a limited spectrum of “solutions” for public policy on many issues. The biggest problems arise when we take our mental models for granted, or they are never consciously updated, and/or they “exist below the level of awareness.”^19^

## So what?

### “Man-made systems become unstable, creating uncontrollable situations even when decision-makers are well-skilled, have all the data and technology at their disposal, and do their best” - Dirk Helbing

So, how do leaders become aware of, understand, and address their biases, mental models, and mindsets in decision and policy-making? Fundamental to this is the need to think about thinking (metacognition). Metacognition is “an awareness of one’s thought processes and an understanding of the patterns behind them”^20^

Systems thinking helps us to understand this further by being aware of our thoughts, emotions, and motivations and, more importantly, how we interact with the outside world. It is, “a particular type of metacognition that focuses on and attempts to reconcile the mismatch between one’s mental models and how the real-world works.”^14^

Metacognition is particularly required in collaborative settings where not only the leader, but each person on the team, must be self-aware, and group-aware to allow *genuine* dialogue and teamwork to emerge—in service of a shared purpose. The “art of not just talking together but of thinking together that seems to have been all but lost in our modern culture.”^21^ Such collaboration needs to work towards a common good. This is characterized as the critical need for “participatory consciousness,” essential to a dialogical approach wherein all the assumptions are put on the table, but suspended, in the group so that thought and meaning can flow easily amongst the group, thus promoting genuine collaboration.^22^

The wicked problems in today’s health system require individuals and silos to work beyond their personal and group agendas towards system level population outcome goals. It is essential that a subsystem’s goals are in harmony with, or in service of, the goals of the larger system or we end up with sub-optimization of the parts^23^ and a tragedy of the commons,^24^ common patterns seen in today’s health systems.

Open minded skepticism is also highlighted as an important “habit of mind” for leaders in complex systems.^25^ The term “epistemic arrogance” describes leaders who make hasty decisions based on wrong or incomplete information and dismissing alternative views, often with highly disruptive or disastrous results. Such leaders need to “adopt a mindset of intellectual humility—an openness to question their own beliefs, seek out diverse perspectives, and accept that their knowledge is finite and fallible.”^26^ Put another way: “That people want to know realities cannot be assumed: strategic ignorance, not wanting to know, is common…. the biggest blind spot of all is ourselves.^27^

Interestingly, this concept of intellectual humility is also felt to be the highest stage of critical thinking as articulated in the stage theory of critical thinking where a master thinker is defined as: “Systematically takes charge of thinking and strives for improvement. Has developed a capacity to intuitively assess thinking for clarity, accuracy, precision, relevance, logic. High degree of intellectual humility, integrity, perseverance, courage, empathy, autonomy, responsibility, and fair-mindedness.”^28^

**Figure 2.**
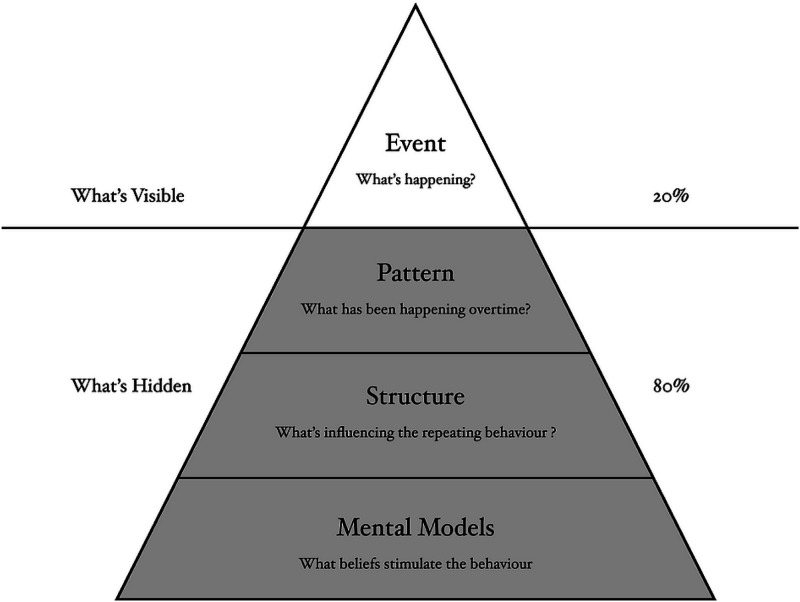
There are similarities between the iceberg model of change and the conceptual framework for creating value in learning health systems.^30^ Unless we are prepared to, and capable of, changing the structures in our healthcare system (and the mental models/mindsets that created them, or resist change to them), we will never be able to change the processes, patterns, and ultimately improve the outcomes in our system.

**Figure 3.**
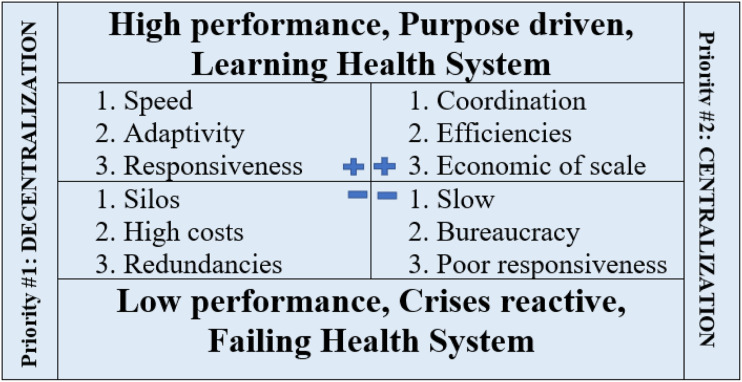
As governments and health authorities consider the benefits and risks of centralization “vs.” decentralization, this polarity management schematic shows how we can balance the best of both (both/and thinking) rather than get stuck in arguing the best of one against the worst of the other (either/or thinking), or worse, pendulum swinging back and forth through major “restructuring” to “fix” the last approach.^32^

## Now what?

### “If you want to understand the deepest dysfunctions in systems, pay attention to the rules, and who has power over them” - Donnella Meadows

Three important mental models/mindsets essential to health leadership are:

**Iceberg model of system change**

***“Every system is perfectly designed to achieve the results that it consistently achieves”* - W. Edwards Deming**

In complex adaptive systems, we must plan for emergence, not outcomes. That is, the patterns that we see, the performance we can measure, and the improved outcomes that we are aiming for, cannot be guaranteed by compliance to a rigidly enforced blue-print plan → Gantt chart timing → execute-the-plan approach to change; rather, they will emerge out of the structures, processes, and relationships that create the system.

An influential paper on the 12 leverage points^15^ of change in a complex system, and where to intervene, ranks them from easiest to implement and least effective, to hardest and most impactful (Figure 2).^29^ The iceberg model of system change simplifies this so we can see how those leverage points relate to each other. The major lesson in this mental model is that the events that we observe in a complex system arise (or emerge) out of our mental models and mindsets—which are the hardest to change, but the most impactful on the system. These mental models then create the structures in the systems, which in turn create the processes and patterns, which in turn gives rise to the outcomes (or performance that we can measure and events we can see).

The iceberg model is relatively easy to understand and it has powerful implications for systems change, but it is harder to implement. It can seem too abstract and not relevant to the day-to-day “real-world” challenges of healthcare practitioner/leaders. It is, therefore, essential that we address those specific “problems” pragmatically, and through a systems lens (when need be—not all problems are wicked). Seeing discrete, local problems as being connected to, dependent upon, and entangled with other problems opens us to more and often counterintuitive leverage points, and more sustainable solutions. There are multiple tools and approaches to aid us in these challenges, many of which are well summarized^8^. Working with these approaches is in itself psychoactive; it helps us to reflect upon and change our own assumptions and mental models.

**Polarity management**

***“Good and bad, I defined those terms, quite clear, no doubt, somehow… but I was so much older then, I’m younger than that now”* - Bob Dylan**

Disagreements create energy. That energy can be used to destroy, or it can catalyze change for the good. Unfortunately, we now live in a very polarized world where differences in thoughts and ideas are turned into emotional responses and are often used as wedges to divide. This is true in society, and it can be true in the design and operations of healthcare systems.

Polarity management is a mental mode/mindset that helps us to adopt a *both/and*, rather than an *either/or* approach to getting the balance right—or at least closer to “the best option” (recognizing there will never be perfect). Rather than letting dilemmas and polarities divide us, we can talk about issues and dialectically work towards optimizing systems, if we can frame these “opposite” perspectives not as either/or, right/wrong, or good/bad choices, but rather as tensions that need to be balanced over time (Figure 3). The question we must ask is: are we dealing with a problem to be solved (for which there really may be one or two right answers), or a tension to be managed (for which we must use polarity management)?

The classic example used to explain polarity management is the inspiration/expiration tension, and which is better for human life?^31^ Breathing in brings in oxygen, which is essential for life, but if there is no time for breathing out, then carbon dioxide accumulates, which is detrimental. Whereas breathing out helps to get rid of CO_2_, but too much breathing out allows no time to breath in O_2_. In a polarized world, one side may argue the benefits of inspiration against the risks of expiration… while the other side will argue the benefits of expiration against the risks of inspiration.

While the inspiration/expiration example may sound simplistic, consider how many healthcare planning exercises degenerate along the lines of one of these polarities: centralization/decentralization, efficiency/capacity, patient rights/responsibilities, and physician autonomy/accountability—to name a few.

**System archetypes**

***“For every complex problem there is a solution that is clear, simple, and wrong”* - Mecken**

Systems archetypes are common patterns that develop in organizations or systems.^33^ They are so common in fact, that they occur over and over again in different systems with different functions—that is why they are called archetypes. Understanding these recurrent patterns of behaviour gives us insight into the drivers and dynamics of systems and how to mitigate against the dysfunctional manifestations that may arise.

At their core, negative system archetypes, or system pathologies,^34^ are what happens in systems when leaders do not adopt the mental models of complex adaptive systems thinking, and instead rely upon mental models more aligned with linear, cause-and-effect, machine-as-system models (Figure 1). All of these archetypes have relevance to health leadership.

One of the most relevant and common archetypes seen in the current Canadian healthcare crisis we are facing is the “fixes that fail” archetype. The dynamics of this system pathology/archetype are seen when the symptom of a deeper problem calls out for a fix; reactive “solutions” are implemented to treat the symptom, leaving the underlying pathology/root cause untouched.^34^ At best, this is a bridge to more structural changes which will eventually reduce the symptoms over time. At worst though, time and resources are rerouted from necessary structural changes, and the symptoms eventually get worse, leading to moral injury in care providers, and tipping points in failed systems over time.^35^

Treating the symptoms of a serious infection is likely to be futile (and potentially catastrophic) if you don’t treat the organism causing the problem, as would hiding the manifestations of a dysfunctional process without fixing the source of dysfunction!

Finally, a change in how we think doesn’t come just because we decide to do it. We need to make an effort to systematically challenge our leadership approach to each demand. Examples of strategies for managing existing cognitive biases, mental models, and mindsets in leadership include those listed below.^5,8,9,11,36-40^

(1) Focus on the co-creating a better system rather than on correcting discrete problems. Consider and monitor impacts of a decision on other parts of the system, in partnership with relevant stakeholders.
(2) Specifically explore whether you are focusing on the “symptom” rather than the “root cause” of the problem—map the system.
(3) Ensure a diversity of perspectives, training, and background in each team (include end users and patients).
(4) Assign devils’ advocates to challenge biases, mental models, and mindsets.
(5) Consider decisions from the point of view of each potential stakeholder, aiming for “both/and,” as opposed to “either/or” solutions, never presuming that you (or your team members) understand the reality or perspective of stakeholders who are not present.
(6) Explore the affective/emotional elements surrounding the problem.
(7) Identify “red flag conditions” such as conflicts of interest, silo interest over systems, and pre-existing attachments.
(8) Identify specific cognitive biases that can threaten rational decision-making in the circumstance and communicate these to the team.
(9) Creative and unrestricted metacognition, “lateral thought,” and “brainstorming exercises” should be encouraged; devalue traditional or accepted principles of problem-solving, questioning what is considered “known” encouraging new perspectives on familiar issues.
(10) Analyze elements of a decision to identify the cognitive processes that led to it—challenge whether a different approach may lead to a different conclusion.

## Conclusion

### What problems in healthcare quality should we target as the world burns around us?

As we ponder the answer to this provocative challenge from a recent editorial^41^ in the CMAJ,^1^ perhaps we need to take a step back. Maybe the better frame for this question is: ***how*** might we address the issues? Our conviction is that we must consciously reflect upon *our biases, mental models, and mindsets*. Better health systems in Canada require a different approach, one that doesn’t just react to the latest event by fiddling around the edges of same old system. We must commit to bold structural reform.^42^ To change our structures, we need mental models and mindsets that are more fit for purpose. Mental models/mindsets don’t change because we wish or will them to. The first step in shifting them is being aware of what they are and how they impact our decision-making. As our sense-making improves, so will our evidence informed leadership and our policy-making, and this will be necessary for meaningful change through true learning health systems.^30^
